# Case of a Boy, Who Recovered after a Cherry-Stone Had Remained Sixty-Eight Days in the Bronchi

**Published:** 1826-11

**Authors:** John Webster

**Affiliations:** Physician to the St. George's and St. James's Dispensary.


### FOREIGN BODY IN THE BRONCHI.

*Case of a Boy, who recovered after a Cherry-Stone had remained sixty-eight Days in the Bronchi.* By JOHN WEBSTER, M.D. Physician to the ST. GEORGE'S and ST. JAMES'S DISPENSARY.

WHEN an extraneous substance is lodged in the trachea or bronchi, besides extraction by the operation of tracheotomy, there are three natural processes, by which it may be removed. It may be expelled by a violent effort of expiration; it may be got rid of by a process of suppuration, in which the abscess bursting externally, the foreign body is allowed to escape between the ribs; or it may be removed by an abscess bursting into the bronchi, and thus, along with the puriform fluid, it makes its escape by the trachea. This latter process was the one which took place in the case now to be detailed.

George R—, fourteen years of age, hitherto in the enjoyment of excellent health, accidentally swallowed a broken cherry-stone. Being at the moment frightened, it slipped into the trachea, when he was suddenly seized with a most violent fit of coughing, accompanied by great difficulty of breathing and a sense of instant suffocation. He had also severe pain, and a slight swelling, at the upper part of the trachea. The dyspnœa, pain, and coughing without expectoration, remained during the night; and next morning he felt as if the foreign body had descended into the chest.

Twelve days after the accident, the patient first came under my care, when he stated that the above symptoms had continued without intermission, and even in a more aggravated form; there was also a constant darting pain under the sternum, extending towards the left side. On sitting up, the difficulty of breathing and sense of suffocation were increased, and he could only lie on the left side.

From examination with the stethoscope, it appeared that the foreign body was situated in the left bronchus, at the bifurcation of the trachea, thereby obstructing almost entirely the passage of air into the left lung. On a further investigation, it was also ascertained that the lung was sound, though collapsed. By percussion on the left side of the chest, a deaf sound was perceived, but in the right cavity every thing seemed healthy. There was a constant palpitation of the heart.

Since the accident, the patient had never expectorated, excepting occasionally a little frothy mucus; and he passed very restless nights, being often affected with violent fits of coughing. Pulse 120, small and sharp; skin warm and moist; tongue clean and red; bowels open, but not regularly so; appetite bad; great thirst; urine scanty and high coloured.

Sixteen leeches, and afterwards a blister, were applied to the chest; besides which, a mixture, containing Squills, Nitrous Æther, and Tartarised Antimony, was given three times a-day, along with three grains of Blue-pill and one of Ipecacuanha.—Very low diet, and absolute rest, were enjoined.

Sixteenth day.—The patient felt considerably relieved by the leeches and blister; but the cough, pain of chest, and laborious respiration, still continue. His nights are very restless, being frequently interrupted by violent fits of coughing, which are never attended with expectoration. Pulse full and quick; skin dry and warm; bowels open.

Was bled at the arm to six ounces; another blister was applied to the chest; and the same medicines continued.

Twentieth day.—For a short time after the bleeding, the patient was better; but now the symptoms are nearly as before. He has but little sleep, and can only lie on his back, feeling as if about to be suffocated when he attempts to move.

Six leeches were applied to the lower part of the sternum, at the left side; and he was ordered the same mixture and pill, with the addition of half a grain of powdered Digitalis to each.

Twenty-second day.—Cough not so violent, but still frequent and coming on in fits; no expectoration, and the difficulty of breathing considerable. Pulse 120, full and rather hard.

Was bled in the arm to eight ounces; the same medicines repeated.

From this time to the sixty-eighth day, the symptoms varied very little, excepting that the patient was sometimes better than at others. The cough, though alleviated, never left him; and latterly he became so weak and exhausted as to be almost unable to move. The pulse continued constantly quick; and he had slight shiverings, indicating the formation of matter.

The stethoscope was frequently employed to ascertain the condition of the thoracic viscera, and on the thirtieth day the following is the report:—" On percussion, and on applying the cylinder, the air appears now to pass more freely into the left lung, particularly into the upper lobe; but the lower one is still further collapsed, which would lead to the supposition that the stone has descended lower into the bronchi, and, by thus freeing one of its branches, the air is allowed to pass into the upper lobe of the left lung with more facility than it did."

The same plan of treatment as at first instituted was pursued; the leeches and blisters were repeatedly applied; and the patient kept exceedingly low and tranquil. Early in the morning of the sixty-eighth day, he felt as if about to be suffocated, with pain extending to the upper part of the neck and left shoulder, followed by sickness and violent fits of coughing, whereby he expectorated more than a pint of fetid pus, mixed with streaks of red blood, and in the midst of which was found the broken cherry-stone. Great exhaustion followed the effort of bringing up so large a quantity of matter, and, for an hour afterwards, life appeared to be nearly extinct. During the day he became more easy, being nearly free from pain, with scarcely any fever or cough: his pulse was ninety-five, and soft; and his skin cool.

A blister was applied to the sternum. Squills, Nitrate of Potash, and Rhubarb, were given every eight hours. He was allowed a little beef-tea, and wine and water, and ordered to remain perfectly quiet.

From the time the stone was expelled, the patient gradually recovered; and, although he spit pus copiously for about a week, he was so well as to be able to walk in the Park on the twentieth day; and, on the ninetieth from the accident, he left town perfectly convalescent.

This case seems to lead to some useful practical conclusions. It shows the importance of diminishing inflammatory action, and of alleviating urgent symptoms, more particularly where it would not be advisable to perform tracheotomy, with a view to extract the foreign body. It also affords an additional and beautiful illustration of one of the processes which nature employs to get rid of an extraneous substance lodged in the bronchi: thus, local inflammation was excited, pus formed around the cherry-stone, and, as soon as a sufficient quantity became collected, the abscess burst, when, by the violent rush of fluid and strong effort of expulsion, the foreign body, the cause of all the mischief, was ejected.

Regarding the propriety of performing tracheotomy in this case, it was the opinion of Dr. Gregory, Messrs. Lawrence, Wardrop, Bacot, and others, who met in consultation, that it would not be an advisable measure; unless the patient should be threatened with impending suffocation, so as to put his life in imminent danger, under which circumstances it ought undoubtedly to be performed: and the successful termination of the case justified the practice which was adopted.

*Grosvenor-street; October 1st*, 1826.

